# Antibiotic associated diarrhea in outpatient pediatric antibiotic therapy

**DOI:** 10.1186/s12887-023-03939-w

**Published:** 2023-03-18

**Authors:** Sevgen Tanır Basaranoğlu, Ayşe Karaaslan, Enes Salı, Ergin Çiftçi, Zeynep Gökçe Gayretli Aydın, Bilge Aldemir Kocabaş, Cemil Kaya, Semra Şen Bayturan, Soner Sertan Kara, Dilek Yılmaz Çiftdoğan, Ümmühan Çay, Hacer Gundogdu Aktürk, Melda Çelik, Halil Ozdemir, Ayper Somer, Tijen Diri, Ahmet Sami Yazar, Murat Sütçü, Hasan Tezer, Eda Karadag Oncel, Manolya Kara, Solmaz Çelebi, Aslınur Özkaya Parlakay, Sabahat Karakaşlılar, Emin Sami Arısoy, Gönül Tanır, Tuğçe Tural Kara, İlker Devrim, Tuğba Erat, Kübra Aykaç, Özge Kaba, Şirin Güven, Edanur Yeşil, Ayşe Tekin Yılmaz, Sevgi Yaşar Durmuş, İlknur Çağlar, Fatih Günay, Metehan Özen, Ener Çağrı Dinleyici, Ateş Kara

**Affiliations:** 1 Department of Pediatric Infectious Diseases, Hacettepe University Faculty of Medicine, Ankara, 06100 Turkey; 2 Department of Pediatric Infectious Diseases, Istanbul Lutfi Kirdar Kartal Training and Research Hospital, Istanbul, Turkey; 3 Department of Pediatric Infectious Diseases, Sanliurfa Training and Research Hospital, Sanliurfa, Turkey; 4 Department of Pediatric Infectious Diseases, Ankara University, Ankara, Turkey; 5 Department of Pediatric Infectious Diseases, Trabzon Karadeniz Technical University, Trabzon, Turkey; 6 Department of Pediatric Infectious Diseases, Antalya Akdeniz University, Antalya, Turkey; 7 Department of Pediatrics, Sanliurfa Training and Research Hospital, Sanliurfa, Turkey; 8 Department of Pediatric Infectious Diseases, Manisa Celal Bayar University, Manisa, Turkey; 9 Department of Pediatric Infectious Diseases, Erzurum Training and Research Hospital, Erzurum, Turkey; 10 Department of Pediatric Infectious Diseases, Saglik Bilimleri University, Izmir Tepecik Training and Research Hospital, Izmir, Turkey; 11 Department of Pediatric Infectious Diseases, Trabzon Kanuni Training and Research Hospital, Trabzon, Turkey; 12 Department of Pediatric Infectious Diseases, Istanbul Zeynep Kamil Women and Children Training and Research Hospital, Istanbul, Turkey; 13 Department of Pediatric Infectious Diseases, Ankara Kecioren Training and Research Hospital, Ankara, Turkey; 14 Department of Pediatric Infectious Diseases, Istanbul Medical School, Istanbul University, Istanbul, Turkey; 15 Department of Pediatrics, Istanbul Acıbadem Atakent Hospital, Istanbul, Turkey; 16 Department of Pediatrics, Istanbul Umraniye Training and Research Hospital, Istanbul, Turkey; 17 Department of Pediatric Infectious Diseases, Konya Training and Research Hospital, Konya, Turkey; 18 Department of Pediatric Infectious Diseases, Ankara Gazi University, Ankara, Turkey; 19 Department of Pediatric Infectious Diseases, Bursa Uludag University, Bursa, Turkey; 20 Department of Pediatric Infectious Diseases, Ankara Yildirim Beyazit University, Ankara, Turkey; 21 Department of Pediatrics, Bursa Acıbadem Hospital, Bursa, Turkey; 22 Department of Pediatric Infectious Diseases, Kocaeli University, Kocaeli, Turkey; 23 Department of Pediatric Infectious Diseases, Ankara Doktor Sami Ulus Women and Children Training and Research Hospital, Ankara, Turkey; 24 Department of Pediatric Infectious Diseases, Hatay State Hospital, Hatay, Turkey; 25 Department of Pediatric Infectious Diseases, Izmir Doktor Behcet Uz Children’s Hospital, İzmir, Turkey; 26 Department of Pediatrics, Ankara University, Ankara, Turkey; 27 Department of Pediatric Infectious Diseases, Istanbul Acıbadem Atakent Hospital, Istanbul, Turkey; 28 Department of Pediatrics, Eskisehir Osmangazi University, Eskisehir, Turkey

**Keywords:** Antibiotic-associated diarrhea, Outpatient clinics, Amoxicillin-clavulanate, Cephalosporins, Phenoxymethyl penicillins, Macrolides

## Abstract

**Background:**

Antibiotic-associated diarrhea is one of the most frequent side effects of antimicrobial therapy. We assessed the epidemiological data of antibiotic-associated diarrhea in pediatric patients in our region.

**Methods:**

The prospective multi-center study included pediatric patients who were initiated an oral antibiotic course in outpatient clinics and followed in a well-established surveillance system. This follow-up system constituded inclusion of patient by the primary physician, supply of family follow-up charts to the family, passing the demographics and clinical information of patient to the Primary Investigator Centre, and a close telephone follow-up of patients for a period of eight weeks by the Primary Investigator Centre.

**Results:**

A result of 758 cases were recruited in the analysis which had a frequency of 10.4% antibiotic-associated diarrhea. Among the cases treated with amoxicillin-clavulanate 10.4%, and cephalosporins 14.4% presented with antibiotic-associated diarrhea. In the analysis of antibiotic-associated diarrhea occurrence according to different geographical regions of Turkey, antibiotic-associated diarrhea episodes differed significantly (*p* = 0.014), particularly higher in The Eastern Anatolia and Southeastern Anatolia. Though most commonly encountered with cephalosporin use, antibiotic-associated diarrhea is not a frequent side effect.

**Conclusion:**

This study on pediatric antibiotic-associated diarrhea displayed epidemiological data and the differences geographically in our region.

## Introductıon

Antibiotic-associated diarrhoea (AAD) is defined as diarrhoea that develops any time from a few hours after the onset of antibiotic therapy to eight weeks following antibiotic cessation [1]. The direct toxic effects of antibiotics on the intestines include altered digestive function secondary to reduced concentrations of gut bacteria or the overgrowth of pathogenic microorganisms [2]. The bacterial diversity of the intestinal lumen is also diminished after the administration of certain antibiotics, and these alterations in the abundance and composition of gut microbiota further lead to the dysfunction of these microbiota [3]. Moreover, the impact seems considerably more long-standing than originally thought. A study of changes in the gut microbiota after antibiotic treatment for *Helicobacter pylori* infections found that gut microbiota were influenced for up to four years post-treatment. [4, 5]. Recent advances in the understanding of the role of gut microbiota have increased awareness of the effects that antibiotics have on the resident microbial ecosystem and how antibiotics can adversely affect patient health. For instance, alterations in faecal microbial diversity have been identified as a reason for marked overall adiposity and dyslipidaemia in obese individuals [6], while atopy and asthma appear to to be associated with an insufficiency of early life exposure to the diverse environmental microbiota [7]. Despite substantial research, a significant need exists for epidemiological data on AAD in paediatric patients in different communities. In this prospective study, we aimed to evaluate the incidence of AAD in children in an outpatient practice using a well-established surveillance system.

## Methods

### Patient enrollment and surveillance system

**Fig. 1:**
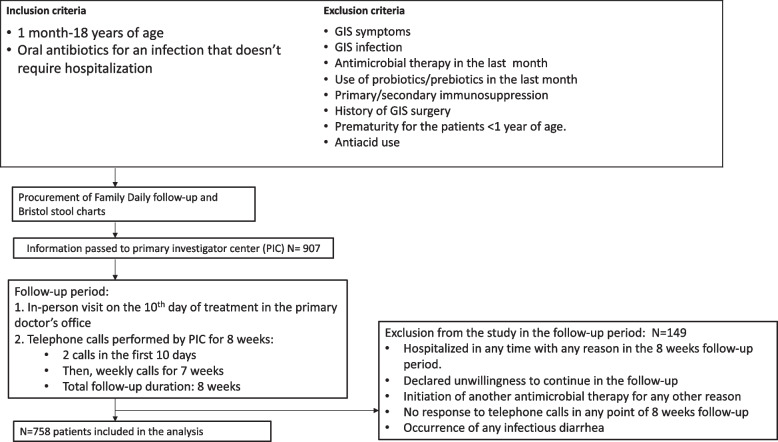
Flow chart of the study, the inclusion, and the exclusion criteria

This prospective multicentre study was performed in Turkey, between October 2016 and August 2018. The primary investigator centre (PIC) was the Department of Pediatric Infecitous Diseases at Hacettepe University Children’s Hospital. The trial was approved by the university’s ethical committee (number: GO 16/487–10). Patients who were between 1 month and 18 years of age, who had started a 10-day oral antibiotic course via paediatric outpatient clinics and for whom we had obtained signed informed parental consent were included in the study. A flow chart of the study and the inclusion criteria were shown in Fig. 1.The initial exclusion criteria were patients who had gastrointestinal symtoms on presentation favoring a gastrointestinal infection, patients who has received antimicrobial therapy and used probiotics/prebiotics in the previous month, the use of antiacids or any type of primary/secondary immunosuppression, a history of gastrointestinal surgery and a history of prematurity in the patients younger than 1 year of age. Cases were included in the study by the primary physician with procurement of family daily follow-up chart (questioning number of daily stool production and appearance) and Bristol stool chart (BSC) [8]. Parents were asked to monitor the number of daily stool production and its consistency in line with BSC. Bristol stool chart comprises a seven point scale, where 1. nuts-like; 2. lumpy sausage; 3. sausage with cracks; 4. smooth snake; 5. soft blobs; 6. fluffy pieces; 7. watery. In the process, the patient had an in-person follow-up visit on the 10^th^ day of treatment in primary physician’s office, and PIC performed a total of 8-weeks follow-up with telephone calls. Two telephone calls in the first 10 days and weekly calls for the next 7 weeks were carried out, during which daily intestinal function was evaluated. Antibiotic associated diarrhea was defined by the daily production of at least three soft or liquid stools (BSC ≥ 4) for at least two consecutive days [9–11]. To confirm that AAD started solely due to the antibiotic therapy as per the defined inclusion criteria, pariticipants were excluded during the eight-week follow-up period if they 1) were hospitalised at any time for any reason; 2) were unwilling to continue with the study; 3) initiated another antimicrobial therapy for any reason (without questioning whether the change was because of diarrhoea); 4) ceased responding to PIC telephone calls; 5) had infectious diarrhoea. If their parents reported that the participant had diarrhoea, the patient was directed to the primary physician to be investigated for an infection or other causes of diarrhoea. In the event of the detection of viruses, bacteria or *Clostridium difficile* as the cause of diarrhoea, the patient was excluded from the study. In addition, if a participant visited their primary physician with gastrointestinal symptoms, their symptoms were evaluated and reported to the PIC to assess the possibility of AAD.

The participants’ demographics, clinical characteristics, the type of infection, the antimicrobial therapy data, AAD occurrence, the time between antibiotic treatment initiation and AAD onset, duration and treatment of AAD, AAD related-hospitalisation, risk factors such as age, geographical region were all collected and evaluated by the PIC.

### Statistical analysis

Statistical analyses were performed using IBM SPSS Statistics (Windows, version 23.0. IBM Corporation Armonk, NY.). Descriptive statistics were used to summarise the participants’ baseline characteristics. These comprised medians and minimum and maximum values for the continuous variables and frequency distributions for the categorical variables. The *p*-values were calculated using the Chi-squared or Fisher's exact tests for the categorical variables and the Student’s *t* or Mann–Whitney U tests for the continuous variables based on the normality assumption. The Kolmogorov–Smirnov test was used to test the normality of the quantitative variables.

## Results

In total, 907 paediatric patients from 28 contributing centres were reported to the PIC. In the eight-week follow-up period, 149 participants were excluded due to hospitalization without diarrhoea or for any reason other than diarrhoea during the follow-up period, an expressed desire to to discontinue their participation, initiation of another antimicrobial therapy, no responses to two or more consecutive telephone calls by the PIC and a diagnosis of infectious diarrhoea. As a result, 758 participants were included in the final analysis (Fig. 1).

The majority of the participants were male (*n* = 396,52.2%). The median age was 4.8 years [IQR: 2.4–8.06), with 19% of the participants between 1 and 24 months, 46.7% were between 2 and 7 years, 23% were between 7 and 12 years, and 11.5% were older than 12 years of age.

**Fig. 2:**
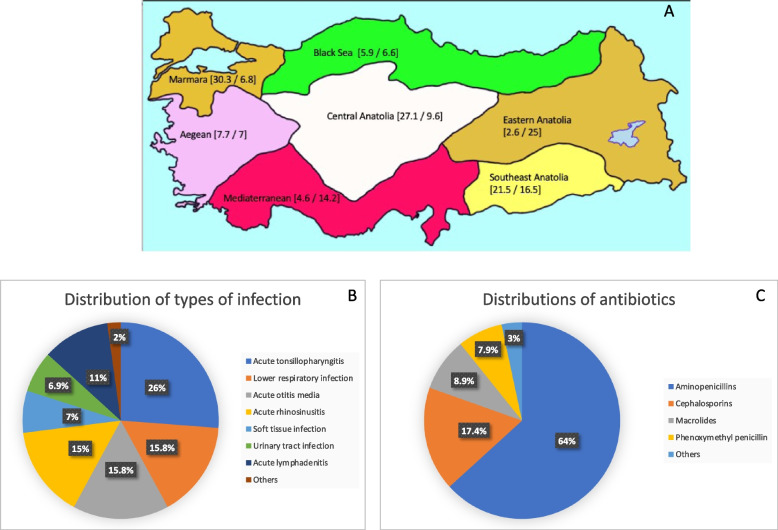
Population characteristics of pediatric cases. **A** Distribution of cases according to geographical regions (percent of cases from each region**/**percent of AAD cases in each region), **B** Types of infections, and **C** Types of prescribed antibiotics

Figure 2 shows the participants’ distribution according to geographical regions, type of infection, and antibiotic groups. The participants were from all seven regions of Turkey: Marmara region, *n* = 230 (30.3.%); Central Anatolia, *n* = 206 (27.7%); Southeast Anatolia, *n* = 163 (21.5%); Aegean region, *n* = 59 (7.7%); Black Sea region, *n* = 45 (5.9%); Mediterranean region, *n* = 35, (4.6%) and Eastern Anatolia, *n* = 20 (2.6%)]. The reasons for antibiotic treatment were acute tonsillopharyngitis (*n* = 198, 26%), lower respiratory tract infection (*n* = 120, 15.8%), acute otitis media (*n* = 120, 15.8%), acute rhinosinusitis (*n* = 114, 15%), acute lymphadenitis (*n* = 84, 11%), soft tissue infection (*n* = 53, 7%), and urinary tract infection (*n* = 52, 6.9%). The most common antibiotic groups prescribed were aminopenicillins (*n* = 486, 64%), cephalosporins (CEP) (*n* = 132, 17.4%), macrolides (*n* = 68, 8.9%) and phenoxymethyl penicillin (*n* = 57, 7.5%).

The characteristics of the participants according to AAD occurrence were displayed in Table 1. During the study period, 79 (10.4%) of the study population presented with AAD. No significant differences were apparent between the participants with and without AAD in terms of age group, type of infections and antimicrobial treatment (*p* > 0.05). In terms of age distribution of participants with AAD, 14.7% were < 2 years of age, 14.9% were > 12 years, 8.8% were 2–7 years, and 6.9% were 7–12 years. A significant difference was evident among the participants with and without AAD episodes based on geographical location (*p* = 0.014). A sub-analysis of the regions showed that those from Eastern Anatolia and Southeastern Anatolia had significantly higher incidences of AAD, compared to the rest of the country (*p* = 0.04). Among the patients treated with amoxicillin-clavulanate (AMC) 10.4% presented with AAD; the same applied to 14.4% of those treated with CEPs, 10% of those treated with phenoxymethyl penicillins, and 6% of those treated with macrolides. Among the participants treated with the usual dose and a high dose of AMC, 8% and 12.3% had AAD in the follow-up, respectively.

**Table 1:** Population characteristics evaluated according to AAD incidence

	Cases w/o AAD *N* = 679	Cases w AAD *N* = 79	*p*
Age (years; IQR)	4.9 (2.6–8.0)	3.5 (1.7–8.9)	
Gender^a^			0.10
Male	360 (53)	36 (45)	
Age groups^a^			0.10
1–24 months	122 (85.3)	21 (14.7)	
2–7 years	323 (91.2)	31 (8.8)	
7–12 years	170 (91.4)	13 (6.9)	
> 12 years	74 (85.1)	13 (14.9)	
Geographic regions^a^			0.014*
Eastern Anatolia	15 (75)	5 (25)	
Southeast Anatolia	136 (83.5)	27 (16.5)	
Mediterranean Region	30 (85.8)	5 (14.2)	
Central Anatolia	184 (90.4)	20 (9.6)	
Marmara Region	215 (93.2)	15 (6.8)	
Aegean Region	55 (93)	4 (7)	
Black Sea Region	42 (93.4)	3 (6.6)	
Type of infection^a^			0.16
Acute tonsillopharyngitis	174 (87.4)	24 (12.1)	
Lower respiratory infection	112 (93.3)	8 (6.7)	
Acute otitis media	103 (85)	17 (15)	
Acute rhinosinusitis	105 (92.1)	9 (7.9)	
Soft tissue infection	45 (84.9)	8 (15.1)	
Urinary tract infection	45 (86.5)	7 (13.5)	
Acute lymphadenitis	78 (92.9)	6 (7.1)	
Others	17 (100)	-	
Treatments and doses^a^			0.48
Aminopenicillins			
Amoxicillin/clavulanic acid (usual dose)^b^	250 (91.6)	23 (8.4)	
Amoxicillin/clavulanic acid (high dose)^c^	142 (87.7)	20 (12.3)	
Amoxicillin/clavulanic acid (max dose)^d^	30 (83.3)	6 (16.7)	
Amoxicillin (high dose)^c^	3 (100)	-	
Amoxicillin (usual dose)^b^	8 (100)	-	
Ampicillin/sulbactam^b^	4 (100)	-	
Cephalosporins	107 (85.6)	18 (14.4)	
Cefuroxime^e^	28 (92.9)	2 (7.1)	
Cefdinir^f^	43 (79.6)	11 (20.4)	
Cefixime^g^	36 (87.8)	5 (12.2)	
Macrolides^h^	64 (94)	4 (6)	
Phenoxymethyl penicillin^i^	51 (89.5)	6 (10.5)	
Others	19 (92.4)	2 (7.6)	

^a^Expressed as percentages; ^b^40-60 mg/kg/day; ^c^80-90 mg/kg/day; ^d^2gr/d; ^e^10-40 mg/kg/d; ^f^14mg/kg/d; ^g^8 mg/kg/d; ^h^clarithromycin: 15–30 mg/kg/d, azithromycin: 10–15 mg/kg/d; ^i^30-80 mg/kg/d

^*^Overall comparison of the regions

**Fig. 3:**
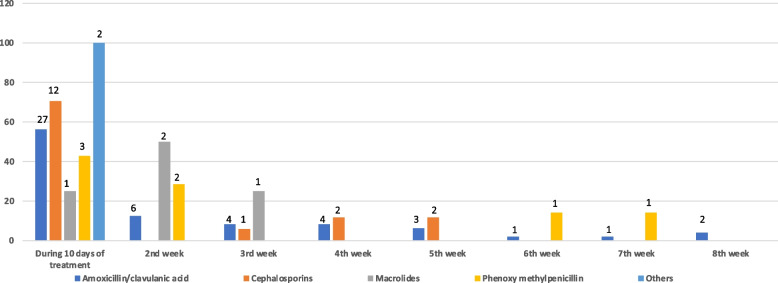
Follow-up of occurence of AAD according to timing of each antimicrobial treatment. The numbers over the bars show the numbers of case who presented AAD

Among the AAD episodes, 56% (*n* = 45) occurred during the 10 day-period of treatment. Figure 3 presents the distribution of AAD cases during the follow-up period. Seventy-percent of AAD cases who were treated with CEPs, 56.2% of AMC, and 42.8% of phenoxymethyl penicillin were observed during the 10 days of treatment. AMC therapy caused diarrhoea in at least one patient in each of the follow-up weeks, while macrolides exerted diarrhoeal side effects in the first three weeks of follow-up. The participants with AAD scoring 7 on the BSC accounted for 63% (*n* = 26) and 76% (*n* = 29) percentage of cases with AAD who displayed with a Bristol scale of 7 were 63% (*n* = 26) and 76% (*n* = 29) of those in the 10-day treatment period and in the following weeks, respectively.

Two patients treated with AMC (dose: 60 mg/kg/d) needed hospitalisation because of AAD during the study period. They were analysed for other infectious causes of diarrhoea and diagnosed with AAD. One was an 18-month-old female admitted with diarrhoea at the fourth week of follow-up and treated with IV fluids for 3 days. The other patient was a 1-year-old male at the third day of antimicrobial treatment. He was hospitalised for 4 days and treated with IV fluids.

## Discussion

In the present study, we analysed the occurrence of AAD in a paediatric outpatient population receiving antimicrobial treatment. The participants were followed up for eight weeks and 10.4% of cases had an AAD episode. There was a wide range of antibiotic classes included in the study and the most common types of antibiotics as the reason for AAD were CEPs and AMC. One of the intriguing findings in the analysis was the significant geographical difference in AAD occurrence.

Antibiotics disarrange intestinal microbiota, thus changing the the diversity and numbers of bacteria in the gut, which leads to AAD. Resident microbiota cannot resist the invasion of pathogenic microorganisms, and endogenous opportunistic pathogens become overgrown [12–14]. This disruption effect may be long lasting, and impact a patient’s susceptibility to other infections [15]. The prevalence of AAD in pediatric outpatient clinics differed between 6.2–62% in many studies from all over the world [16–20], with no data from Turkey. The incidence of AAD in outpatient pediatric clinics was reported as 6.2% and 11% in population studies, and 14% in clinical trials [1]. To the best of our knowledge, our study gives data from the longest surveillance of AAD in paediatric population treated with oral antimicrobials and we found 10.4% of AAD prevalence of AAD in our population. Therefore, the percentages in the present study was similar to previous results. In a US data set [10], younger age was a risk factor for AAD development, whereas a Thailand study [16] could not demonstrate an association between different ages. Similarly, the present analysis of the age groups did not reveal any differences. Notwithstanding, patients older than 12 years and those younger than 2 years had the same frequency of AAD, which was relatively higher than that of the other age groups. Yassour et al. showed the negative effect of frequent antibiotic use on infant microbiome diversity: children younger than 3 years with repeated antibiotic exposure had a less stable intestinal microbial composition, and their gut microbiomes presented with antibiotic resistance genes after treatment [20]. Likewise, changing dietary patterns in adolescence may be a causal factor for diarrhoea, as evidence shows different gut microbiomes in adolescents with vegetable -based and meat -based diets [21].

In terms of risk factors for the occurence of AAD in outpatient clinics, Truck et al. [10] found that the incidence of AAD was particularly high after AMC administration, while Crew et al. showed that recent CEP use was an important risk factor for community-acquired *Clostridium difficile* diarrhoea [22]. In the present study, among the cases treated with CEPs, AAD occurred relatively more frequently in patients treated with CEPs than those who used AMC, with the prevalence over 10% percent for both groups. A recent systematic review of antibiotic induced changes in gut microbiota found that the administration of amoxicillin caused an overgrowth of *Enterobacteria*, whereas changes in the anaerobic population including *Lactobacillus*, *Bifidobacterium* and *Bacteroides* differed tremendously between studies from no change, to increases and/or decreases, as well as changes in diversity [23]. Additionally, administration of AMC caused an increase in *Enterobacteria* with divergent effects on anaerobic bacteria, *Bifidobacterium* sp, *Lactobacillus* sp and *Bacteroides* sp and a general decrease in diversity. Some studies have shown that these effects may last as long as 3–4 weeks [24, 25]. Many changes in microbiota caused by cephalosporins have been noted in the literature. Most studies using cephalosporins have reported a decreased abundance of *Enterobacteriacaea;* however, a few have documented an increase [26]. The abundance of *Bacteroides* spp. increased with the use of first- and second-generation cephalosporins, but decreased with higher generation cephalosporins. An analysis of other antibiotics, such as ciprofloxacin, minocycline, clindamycin, paromomycin and clarithromycin plus metronidazol, revealed further devastating effects of antibiotics on bacterial diversity [26]. The duration, dosing of antibiotics and pre-treatment microbiota composition may influence the changes in the gut microbiome and their duration and the occurrence of diarrhoea [27, 28].

An intriguing finding of our study was that the frequency of AAD differed significantly between the geographic areas in Turkey, which may be due to the well-known nutritional factors differ between regions. Nutritional habits differ between the east, south and west of the country. For example, food in east and southeast of Turkey comprises red meat and a high fat content. In contrast, on the south and west coasts, the food is mainly a Mediterranean style. Our study results bring to mind the question of the relationship between nutritional factors and AAD occurence. The recent data showed that a high fat high protein diet was associated with a dysbiotic microbiome and metabolome in the colon [29, 30], while a high fat diet reduced *Akkermansia municiniphila* and *Lactobacillus* which are related to healthy metabolic states [31]. In a discussion on the relationship between the gut microbiome and diet, although high animal protein consumption was found to be positively associated with overall microbial diversity, a contrary result of lower short-chain fatty acids was detected in children with high animal protein diets [32]. It is well known that microbial-derived short-chain fatty acids contribute to lower inflammatory responses [33]. Similar to the discussions about protein/fat content of nutrition and gut microbiom, effect of high carbohydrate content of nutrition was investigated as well. Digestible and non-digestible carbohydrates were reported to augment *Bifidobacterium* and decrease the amount of *Clostridia*. Non-digestible carbohydrates also increased the amount of *Lactobacillus, Ruminococcus*, *Eubacterium* and *Roseburia*, which displayed a healthy gut microbiome [32, 34–36]. Although research is still needed to clearly define the effects of nutrition on microbiota and the occurrence of AAD, the differences in the types of nutrition in different regions of Turkey may be a factor in our results.

Antibiotics may cause gastrointestinal side effects following the cessation of treatment. In piglets that were given amoxicillin one day after birth, the microbiota remained substantially altered five weeks after treatment, which further emphasises the long-term effects of antibiotics on gut homeostasis [37]. The therapeutic doses that are used in clinical practice do not typically reduce the total bacterial biomass in the gastrointestinal system, but can eliminate the subsets of the microbiota, thus shifting the community in ways that promote colonisation by opportunistic pathogens [38]. In the present study, the close follow-up and observation of patients with AAD by the particular PIC provided a reliable and well-controlled form of surveillance. Moreover, we found that the incidence of AAD was independent of the site of the infection and the choice of antibiotics. It was shown that AAD may be observed until the end of surveillance, with most of the agents causing AAD in the first 10 days of treatment. Amoxicillin-clavulanate and phenoxymethyl penicillin gave rise to the most extended diarrhoeal effects in our outpatient follow-up. With the exception of two, most of the cases were self-limited.

This study had some limitations. First, we were not able to analyse the stools and define changes in microorganisms in the AAD cases. Second, the effects of probiotics were not tested in the study. Third, despite a quite reasonable time of follow-up period, the number of participants could have been higher if we had examined the impact of a wider range of antibiotic groups. Finally, we did not assess the rate of diarrhea with other causes among patients in different regions of the country who did not receive antibiotics as a comparator group. Accordingly, the baseline rates of diarrhoea were not evaluated in our study.

In conclusion, the study presents the data on the occurrence of AAD in paediatric outpatient care with a valuable time of follow-up period. It provides some clues regarding the epidemiological differences in AAD occurence, which may inform clinicians for anticipating AAD in their own region. This may help them make more meticulous decisions regarding antibiotic prescriptions, even to the extent of performing better antimicrobial stewardship practices. Further analysis in conjunction with microbiata milieu may highlight the geographical differences in AAD occurence. Importantly, the our data revealed the potential duration of antibiotics’ gastrointestinal effects in a paediatric outpatient population, which may avoid from precribing antibiotics in unnecessary clinical states, especially in children.

## Data Availability

The datasets generated and/or analysed during the current study are not publicly available due to the fact that the trial participants are children and parental consent was obtained. But are available from the corresponding author on reasonable request.

## Abbreviations

AAD: Antibiotic-associated diarrhea
PIC: Primary Investigator Center
BSC: Bristol stool chart
AMC: Amoxicillin-clavulanate
CEP: Cephalosporin
